# Relationship between psychosocial working conditions, stress perception, and needle-stick injury among healthcare workers in Shanghai

**DOI:** 10.1186/s12889-019-7181-7

**Published:** 2019-07-04

**Authors:** Chao Wang, Li Huang, Jue Li, Junming Dai

**Affiliations:** 1 School of Public Health, Fudan University, Shanghai, China; 2 Department of Disease Surveillance, Beijing Prevention and Treatment Hospital of Occupational Disease for Chemical Industry, Beijing, China

**Keywords:** Needle-stick injury, Stress perception, Psychosocial working conditions

## Abstract

**Background:**

The present study aims to identify the association between psychosocial working conditions, global stress perception, and needle-stick injury among Chinese healthcare workers. It also endeavors to detect the mediating effects of global stress perception.

**Methods:**

A total of 1956 valid samples were collected from eight teaching hospitals in Shanghai, China. A self-reported questionnaire was administered to participants after obtaining their written consent. Structural equation model was used to analyze the relationship between study variables.

**Results:**

Most of the correlation coefficients between psychosocial conditions at work, stress perception, and needle-stick injury are of statistical significance ranging from 0.004 to 0.869. Results of the internal consistency test shows that Cronbach’s α is between 0.770 and 0.925. All three models for effect analysis demonstrated satisfactory global goodness and acceptable path loadings. Psychosocial working conditions and stress perception were directly associated with events of needle-stick injury, as 0.39 (95%CI: 0.32 to 0.48) and 0.32 (95%CI: 0.22 to 0.39), respectively. Furthermore, stress perception had been proved to have a mediating effect (0.25, 95%CI: 0.19 to 0.31) between psychosocial working condition and needle-stick injury, which occupied over one-third of the total effect.

**Conclusions:**

Both stressful psychosocial working conditions and negative stress perception could increase the risk of needle-stick injury that occurs among healthcare workers. Management of stress perception could reduce health risk brought by stressful psychosocial working conditions.

**Electronic supplementary material:**

The online version of this article (10.1186/s12889-019-7181-7) contains supplementary material, which is available to authorized users.

## Background

Needle-stick injury happens when sharp instruments such as a needle penetrates the skin during medical practices. If the sharp instrument is contaminated with blood and bodily fluids, there is potential for transmission of infectious disease [1, 2]. needle-stick injuries are a serious occupational hazard in the healthcare profession, and the distribution of risk does not occur at random [3]. Thus, needle-stick has aroused much attention among medical experts during the past years [4, 5]. Sharps injuries are popular hidden problems among health care workers; after the incidence happens, most medical practitioners neglect their injury and continue with their work [5]. In China, the compliance of wearing gloves when handling contaminated needles among healthcare is at a low level, and Hui reports that 78.9% of nurses in China do not wear gloves when required, which is likely due to the lack of knowledge and positive attitudes toward preventing sharp injury [6]. The main reasons for not reporting sharp injuries are as follows: little or no perceived risk, embarrassment, lack of time, fear of post-reporting results (impact on future careers, being blamed by other classmates, teachers, etc.), reluctance to admit ignorance about how to deal with equipment, and lack of knowledge about how or where to report [7]. Neither the magnitude of the risk of needle-stick injury nor the practices (such as medical operation with nonretracting finger-stick lancets and glass capillary tubes) associated with it have been well understood by healthcare workers [8]. As a matter of fact, exposure to blood and other body fluids via contaminated needle-sticks and sharp devices is a significant occupational hazard, potentially leading to infection with blood-borne pathogens among healthcare workers [9]. Each year, more than three million healthcare workers are exposed to Hepatitis B Virus (HBV), Hepatitis C Virus (HCV), and Human Immunodeficiency Virus (HIV) due to sharp injuries [10]. Previous studies have shown that, out of every six needle-sticks, one person is infected with Hepatitis B., out of every 10 injuries, one person is infected with Hepatitis C Virus, and out of 300 injuries, one person is infected with HIV [11].

Needle-stick incidents are associated with a number of different job factors, including heavy workload, working in surgical or intensive care units, insufficient work experience, and young age [12]. Unfortunately, most previous studies concerning the relationship between psychosocial working conditions and sharp injury have primarily treated sharp injury as a stressor of psychosocial conditions at work, while neglecting the influence of stressful condition on the high incidence of sharp injury at work [13, 14]. Furthermore, few studies have addressed psychosocial working conditions as a factor in sharp injury. Gholami’s study shows that the number of shifts a healthcare worker works per month, which is typically a kind of risk psychosocial condition, was found to be significantly associated with occurrence of needle-stick and sharps injuries [13]. Yonezawa also posits that working at night might significantly increase the risk of sharp injury among doctors using used general anesthesia [15]. Factors such as working in a highly stressful industry, long working hours, increasing administrative workload, broader responsibilities, irregular rhythms of life, verbal and physical abuse by patients, to name a few, characterize the psychosocial working conditions of healthcare workers and risk their psychological health [16]. Studies have shown that levels of dissatisfaction, work stress, and burnout at work are high among healthcare workers and may even be higher than workers of other occupations [17]. Thus, discovering the role of stressful working conditions in the development of sharp injury at work is essential in occupational health studies of healthcare workers [16]. Long-term exposure of stressful working conditions may result in fatigue, sleep disorders, burnout, anxiety, among others, and will further lead to impaired satisfaction, poor performance, and attention-deficit, which are important predictors of safety behavior and negative events at work [18]. Therefore, this study explores the relationship between stressful working conditions and needle-stick injury to detect any direct or indirect correlation between them, in order to provide scientific evidence on strategies for the prevention of workplace injury.

Little exploration has been done on how stressful psychosocial working conditions influence health by affecting the stress perception with the relevant individual [19]. Practically, stressful psychosocial working conditions are proved to be highly correlated with stress perception. Many studies have explored the relationship between them, such as Wallgren and Hanse, who surveyed 167 information technology consultants in Sweden and found that job demand is positively related to perceived stress [20]. Vanagas and Bihari Axelsson found that high job demand associated with low job decision latitude have the greatest impact on stress among 300 Lithuanian General Practitioners [21]. Bin Nordin Rusli pointed out that high social support reduces self-perceived stress [22]. In other words, stressful working condition is not equal to perceived stress that may perform different effects on psychological or physical health [23]. It may generate different interpretations, responses, and coping strategies among individuals with different experiences, personality traits, personal goals, working expectations, standards, and concerns [22].

Considering the fact that improving working conditions is not always readily available, strategies to promote health should also focus on the perceptions employees have of their stress. More specifically, we assume that interventions into stress perception might be helpful in preventing needle-stick injury among healthcare workers when the working environment is difficult to change. One would argue that healthcare workers with more optimistic perceptions of stress would experience fewer needle-stick injuries under certain high-risk working conditions. It is then necessary to identify the relationship between psychosocial environments and perceived stress, effect of stressful working conditions on needle-stick injury occurrence, and the role of stress perception act between psychosocial working conditions and needle-stick. However, the extent of the problem and factors associated with its occurrence are limited in the study area. The study intends to clarify the interaction between environment-individual cognition-behavioral changes, by adopting the analysis of structural equation model to test the following three hypotheses: 1) higher-risk psychosocial working conditions directly relate to more serious stress perceptions and more incidents of needle-stick injury; 2) a more serious stress perception directly relates to more incidents of needle-stick injury; 3) stress perception can mediate the relationship between psychosocial working conditions and needle-stick injury.

## Methods

### Involvement of study population and investigation process

The study recruited employees from eight teaching hospitals in Shanghai, China, using a cross-sectional survey. Using simple random sampling, eight of 20 teaching hospitals in Shanghai were selected for participation in this study, and proportional to size sampling was used for the incorporation of study participants according to different number and categories of healthcare workers within each hospital selected.

Sample size was calculated through the following equation, in which α (α error) =0.05, *δ* (tolerance error) =0.022, *p* (prevalence of needle-stick injury among healthcare workers) =0.45. Considering that 10% of the samples were invalid, some 2200 samples were included in the study.

$$ n={\left[\frac{u_{\alpha }}{\sin^{-1}\frac{\delta }{\sqrt{p\kern0.5em \left(1-p\right)}}}\right]}^2 $$

The field survey was directed by the School of Public Health of Fudan University and Beijing Prevention and Treatment Hospital of Occupational Disease for Chemical Industry united with Shanghai medical labor union. Upon consent of the local unit, we conducted on-site mobilization. Field investigation was officially conducted from June 2015 to July 2015. All potential participants were required to meet the inclusion criteria before completing the field survey: (1) participants had worked continuously for 6 months or more in their current healthcare position; (2) participants did not have a history of mental illness and no history of psychotropic drug use for 1 week before the investigation; (3) participants completed on-site mobilization and voluntarily participated in the survey after providing informed consent. All participants who consented to the study answered a series of questionnaires comprising questions on socio-demographics, health, and cognition. All investigators had accepted unified training and were located at each field survey site. Participants completed the questionnaires (see Additional file 1) without the influence of their informants, even in cases when participants had cognitive deficits. Ethical clearance was obtained from the Medical Ethics Committee of the School of Public Health at Fudan University (IRB # 2012-03-0323).

### Measurement

#### Demographic characteristics

Three demographic characteristics were measured as the control variables, including gender (1 = male, 2 = female), age group (1 = 30 years old or below, 2 = 31 to 40 years old, 3 = 41 years old or above), and job position (1 = physician, 2 = nurse, 3 = technician).

#### Psychosocial working conditions

In recent decades, two theoretical models measuring stressful psychosocial work environment have been developed and tested with particular intensity: the job demand-control-social support model (DCS) and the model of effort-reward imbalance (ERI). The well-known DCS model assumes that exposure to work places with a specific task profile (high demands in combination with low control) contributes to stress-related health risk and disease [24]. The ERI model maintains that lack of reciprocity between efforts spent and rewards received (that is, high “cost”/low “gain” conditions) in the work role define a state of emotional distress with special propensity to health risk [25]. The DCS model focuses on the situational aspects of the psychosocial working conditions, whereas the ERI model includes both extrinsic (situational) and intrinsic (person) characteristics. A combination of information derived from the two models may capture a broader range of stressful experiences at work and, thus, result in an improved risk estimation of stress related health effect onset. Moreover, both DCS and ERI model were established on clear framework, which is advantageous for the following strategies of intervention.

The DCS model was administered to assess three dimensions of working conditions: job demand, job control, and social support. A modified Job Content Questionnaire (JCQ, Chinese version) [24], which has been confirmed to have high reliability and validity, was used for the field survey [24]. The Job Content Questionnaire (Chinese version), which has been widely adopted in China, includes 16 items, of which five items assess job demand, six items assess job control, and five items assess social support. All 16 items use the Likert 5-Point Scale answer format, in which a score of “5” means “completely agree” and a score of “1” means “not at all” [24, 26]. Cronbach’s alpha coefficients for total score, job demand, job control, and social support scales were 0.835, 0.885, 0.743 and 0.863, respectively.

The Effort-Reward Imbalance questionnaire (ERIQ, Chinese version) was used to evaluate the other three working conditions: job effort (item 1 to 6), job reward (item 7 to 17), and over-commitment (item 18 to 22) [25]. The answer format of the ERIQ is similar to that of JCQ [25], and both of the two questionnaires have been widely used in occupational stress research. Cronbach’s alpha coefficients for total score, job effort, reward, and over-commitment scales were 0.920, 0.925, 0.882 and 0.948, respectively.

#### Stress perception

The variables of stress perception were evaluated by the Chinese Perceived Stress Scale (PSS-10) and included three dimensions: perceived helplessness (comprised of item 1, 2, 3, 6, 9 and 10, which are negatively worded), perceived self-efficacy (items 4, 5, 7, 8, which are positively coded), and the total stress perception by 10 measuring items. The items are rated according to a 5-point Likert answer format (1 = never to 5 = very often). When computing the total score, the four perceived self-efficacy items are reversely coded and then added to the six perceived helplessness items, so that total score denotes the whole dimension of perceived stress [27]. Chrobach’s alpha coefficients for total score, perceived helplessness and perceived self-efficacy scales were 0.888, 0.806, and 0.770, respectively.

#### Needle-stick injury

The prevalence of needle-stick injury was measured by a single quantitative question restricted to the past one year: “During the past one year, how many times did you experience a needle-stick injury?”

### Statistical analyses

The variable of needle-stick injury was first dichotomized to test differences between demographic groups using the chi-square test, and the study hypotheses were analyzed on the basis of the continuous variable of needle-stick injury. To test if the selected measurement of variables was reliable enough to support the analyses of study hypotheses, we introduced Cronbach’s α to test their internal consistency reliability. Single-factor correlation analysis based on spearman correlation coefficient was preliminarily conducted to explore relationships between psychosocial working conditions, stress perception, and needle-stick injury. Then, the structural equation model was applied to establish the analysis structure of research variables based on the study hypotheses. For global goodness of fit, the adjustment fitting goodness indicator (AGFI), non-normalized fit index (NNFI), incremental fit indicator (IFI), and root mean square error of approximation (RMSEA) were employed. Studies have shown that the model fit coefficient > 0.9, and RMSEA < 0.08 can be accepted as good fit [28]. In this study, the six psychosocial working conditions and needle-stick injury were included and analyzed as measurement variables, and psychosocial working conditions as a whole, stress perception, and the other two sub-dimensions were structured as latent variables. To explore different structural associations of demographic characteristics, we used the hierarchical method of structural equation model. We adopted the method of Nested Model Comparisons to discuss influences of different demographic characteristics on the model’s goodness of fit. Then, we analyzed the model’s path coefficients according to different demographic characteristics. Mediating effect of the structural equation model was also conducted to analyze the direct and indirect effect of study variables on needle-stick injury [29]. The bootstrap statistical method, which was recommended and powerful in the calculation of mediating effects, was conducted [30, 31]. The resampling number was set as 2000, according to Hayes, taking the bias-correction interval as the confidence intervals (CI) of mediating effect [32]. EpiData3.1 was used for data entry, and IBM SPSS Statistics for Windows (Version 22.0) and IBM SPSS AMOS (Version 21.0) were used for statistical analysis; α takes 0.05 with two tails.

## Results

### Characteristics of study participants

A total of 2200 questionnaires were issued in the survey, and 1956 (88.91%) valid samples were eligible for the analysis. Among the whole sample, 28.58% of respondents were male, and 71.42% were female. The distribution of participants according to age group was relatively homogeneous: 655 (33.49%) were 30 years old or younger, 713 were at the age group of 31 to 40 years old, and 588 were 41 years old or older. Moreover, 618 (31.60%) of respondents worked as physicians, 842 (43.05%) were nurses, and 496 (25.36%) were medical technicians. The chi-squared test showed that the distribution of gender, age, and job position between two groups of needle-stick injury were of statistical difference (*p* < 0.05). Young female nurses were at higher-risk of needle-stick injury. The average frequency of needle-stick injury was 1.19 ± 1.75 (ranges between 0~9 times) and incidents of needle-stick injury among employees were recorded at least once showed to be 2.58 ± 1.76 ranging between 1 to 9 times (see Table 1 for more details).

**Table 1:** Characteristics of participants in the study

Variable	Whole sample	Needle-stick n (%)		x^2^	*p-*value
	n(%)	Not happened	≥1 time/year		
Whole sample	1956(100.00)	1056 (53.99)	900 (46.01)	–	–
Gender					
Male	599 (28.58)	334 (59.75)	225 (40.25)	10.46	.001
Female	1397 (71.42)	722 (51.68)	675 (48.32)		
Age group					
≤ 30 yrs.	655 (33.49)	295 (45.04)	360 (54.96)	74.42	<.001
31–40 yrs.	713 (36.45)	360 (50.49)	353 (49.51)		
≥ 41 yrs.	588 (30.06)	401 (68.20)	187 (31.80)		
Position					
Physician	618 (31.60)	323 (52.27)	295 (47.73)	206.73	<.001
Nurse	842 (43.05)	335 (39.79)	507 (60.21)		
Technician	496 (25.36)	398 (80.24)	98 (19.76)		

### Analysis of correlation and internal consistency of variables

As is shown in Table 2, most of the correlation coefficients between psychosocial working conditions, stress perception, and needle-stick injury are of statistical significance ranging from 0.004 to 0.869. Needle-stick is positively correlated to job demand, job effort, over-commitment, helplessness, low self-efficacy, and total stress perception, 0.400, 0.500, 0.450, 0.538, 0.191, and 0.528, respectively (*p* < 0.05). In addition, job control, social support, and job reward are negatively correlated with needle-stick injury, − 0.153, − 0.169 and − 0.424, respectively (*p* < 0.05). The correlation between total stress perception and job demand, control, support, effort, reward and over-commitment are of statistical significance, 0.318, − 0.193, − 0.339, 0.521, − 0.530, 0.515, 0.869, and 0.629, respectively (*p* < 0.05), while the coefficient between job demand and low self-efficacy has no statistical significance as 0.004 (*p* > 0.05). In addition, results of the internal consistency test show that Cronbach’s α is between 0.770 and 0.925. Among these, the reward dimension has the highest reliability (0.925), total stress perception has the lowest (0.754), and the internal consistency of remaining dimensions are all above 0.800, thus reaching a high level (see Table 2 for details).

**Table 2:** Correlation between variables in the study

Variable	Cronbach’s α	1	2	3	4	5	6	7	8	9	10	11	12
1.Gender	–	1.00											
2.Age group	–	−0.159^**^	1.00										
3.Position^a^	–	−0.368^**^ 0.525^**^	0.185^**^ 0.322^**^	1.00									
4.Job demand	0.885	0.041	−0.099^**^	0.105^**^ 0.158^**^	1.00								
5.Job control	0.743	−0.119^**^	0.116^**^	0.157^**^ −0.139^**^	− 0.066^**^	1.00							
6.Social support	0.863	0.005	0.039	0.037 −0.045^*^	0.128^**^	0.316^**^	1.00						
7.Job effort	0.920	0.006	−0.140^**^	0.122^**^ 0.170^**^	0.631^**^	−0.111^**^	−0.098^**^	1.00					
8.Job reward	0.925	0.049^*^	0.055^*^	−0.050^*^ 0.085^**^	−0.315^**^	0.180^**^	0.399^**^	−0.574^**^	1.00				
9.Over-commitment	0.882	0.008	−0.084^*^	0.070^**^ 0.173^**^	0.475^**^	−0.069^**^	−0.183^**^	0.682^**^	−0.569^**^	1.00			
10.Helplessness	0.888	0.062^**^	−0.115^**^	0.037^**^ 0.144^**^	0.418^**^	−0.188^**^	−0.249^**^	0.589^**^	−0.501^**^	0.563^**^	1.00		
11.Low self-efficacy	0.806	0.058^**^	−0.163^**^	0.093^**^ −0.163^**^	0.004	−0.126^**^	− 0.299^**^	0.128^**^	− 0.251^**^	0.151^**^	0.224^**^	1.00	
12. stress perception	0.770	0.066^**^	−0.172^**^	−0.023 0.197^**^	0.318^**^	−0.193^**^	− 0.339^**^	0.521^**^	− 0.530^**^	0.515^**^	0.869^**^	0.629^**^	1.00
13.Needle-stick injury	–	0.073^**^	−0.190^**^	0.015 0.258^**^	0.400^**^	−0.153^**^	−0.169^**^	0.500^**^	−0.424^**^	0.450^**^	0.538^**^	0.191^**^	0.528^**^

Note:*: *p* < .05; **: *p* < .01; Double tailed for all tests; ^a^: variable of position has been analyzed by 2 dummy variables, as “0, 0” = technician, “1, 0” = physician (list ahead), “0, 1” = nurse (list behind)

### Establishment and optimization of structural models

By confirming that the selected variables’ correlation and internal consistency permits the model fitting, we then conducted structural data fitting and optimization under the hypotheses of the study which were statistically verified. In the first place, psychosocial working condition model and stress perception model were structured separately. Local indices show that the global fit of the modified models is more acceptable compared with their original model: CMIN/DF = 24.77, AGFI = 0.912, NNFI = 0.913, IFI = 0.971, RMSEA = 0.063 for the model of psychosocial conditions at work; CMIN/DF = 5.06, AGFI = 0.972, NNFI = 0.979, IFI = 0.987, RMSEA = 0.046 for the model of stress perception. In the M3 model, we structured psychosocial working conditions, helplessness and needle-stick injury together to test if there were significant correlating effects under study hypothesis. Results show that the M3 model achieves high global goodness of fit, as CMIN/DF = 7.52, AGFI = 0.946, NNFI = 0.960, IFI = 0.971, RMSEA = 0.058 under the modified model. Similarly, the psychosocial working conditions-low self-efficacy-needle-stick model shows satisfied goodness-of-fit, CMIN/DF = 11.34, AGFI = 0.932, NNFI = 0.926, IFI = 0.949, RMSEA = 0.073. Finally, the psychosocial working condition-stress perception-needle-stick model was structured whose model goodness of fit remains satisfied (CMIN/DF = 7.07, AGFI = 0.936, NNFI = 0.946, IFI = 0.958, RMSEA = 0.056), and the coefficients of each path are each statistically significant (see Table 3 for details).

**Table 3:** The process of model fitting and the goodness of fit index of structural models

dimensions/models		x^2^	df	x^2^/ df	AGFI	NNFI	IFI	RMSEA
M1.	Original	821.74	9	91.30	0.689	0.670	0.802	0.215
	Modified	123.83	5	24.77	0.912	0.913	0.971	0.063
M2.	Original	2852.55	35	81.50	0.583	0.577	0.671	0.203
	Modified	136.62	27	5.06	0.972	0.979	0.987	0.046
M3.	Original	1441.80	63	22.89	0.840	0.864	0.890	0.106
	Modified	428.41	57	7.52	0.946	0.960	0.971	0.058
M4.	Original	1155.22	42	27.51	0.824	0.810	0.855	0.116
	Modified	430.91	38	11.34	0.932	0.926	0.949	0.073
M5.	Original	4211.74	117	36.00	0.677	0.689	0.733	0.134
	Modified	742.31	105	7.07	0.936	0.946	0.958	0.056

Note: M1: the fitting model of psychosocial working conditions only; M2: the fitting model of total stress perception measurement; M3: the model includes psychosocial working conditions, dimension of helplessness and needle-stick; M4: the model includes psychosocial working conditions, dimension of low self-efficacy and needle-stick; M5: the model includes psychosocial working conditions, stress perception and needle-stick

### Path coefficient of structural equation model

The paths of the M3 measurement model have acceptable loadings, among which the standardized regression weight ranges from 0.64 to 0.83 of helplessness dimension and 0.17 to 0.84 of psychosocial working condition dimension. Among the latent variables, the standardized regression weights of psychosocial working condition to helplessness, psychosocial working condition to needle-stick injury, and helplessness to needle-stick injury are 0.78, 0.40, and 0.31, respectively (*p* < 0.05). Then a hierarchical method of structural equation model was used to detect the multi-group difference between gender, age, and position. Results show that age or position influences the regression effects: for those participants belonging to the age group of 41 years old or older, the latent variable of psychosocial working condition shows less effect on the feeling of helplessness and needle-stick injury, which are 0.69 and 0.32 (*p* < 0.05), respectively; helplessness causes a greater effect on needle-stick injury for participants aged 31 to 40 years old at 0.38; medical technicians show less regression weight from psychosocial working conditions to helplessness at 0.66 (*p* < 0.05), less working condition-needlestick effect 0.22 (*p* < 0.05), and higher helplessness-needlestick effect 0.33 (*p* < 0.05) compared to the other two position groups.

Similarly, the paths of the M4 measurement model vary from 0.15 to 0.87, which shows satisfied internal consistency. Standardized estimates on the path coefficient still have statistical significance, while the effects are of much difference compared to the M3 model. Regression weights from psychosocial working conditions to low self-efficacy and needle-stick injury are 0.10 and 0.63 (*p* < 0.05), respectively, and low self-efficacy to needle-stick injury is 0.06 (*p* < 0.05). For the multi-group analysis, compared to the other two age groups, 31 to 40 years old employees have a greater influence on the path from psychosocial working conditions to low self-efficacy 0.16 (*p* < 0.05) and psychosocial working conditions to needle-stick injury 0.66 (*p* < 0.05), while the regression weight from low self-efficacy to needle-stick injury is not statistically significant at 0.01 (*p* > 0.05). The psychosocial working condition-low self-efficacy path presents much higher coefficient as 0.21(*p* < 0.05) among physicians compared to nurses (0.03, *p* > 0.05) or medical technicians (0.09, *p* < 0.05).

**Fig. 1:**
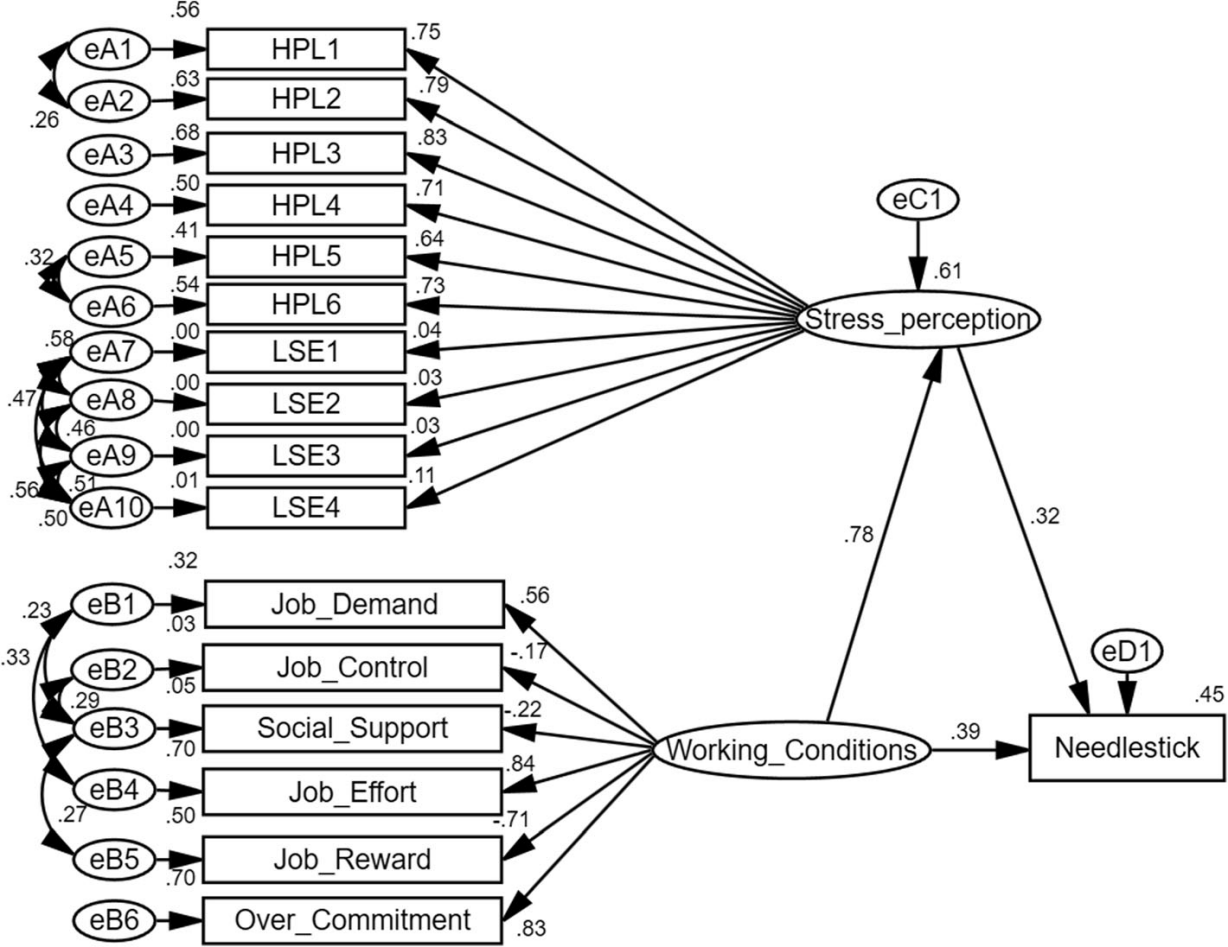
Structural Equation Model was conducted within study variables: psychosocial working condition, stress perception, and needle-stick injury

For the M5 model, helplessness and low self-efficacy were both intended to test the whole effect between psychosocial working conditions, stress perception, and needle-stick injury. As shown in the M5 measurement model, the standardized regression weight ranges from 0.03 to 0.84, among which the items of low self-efficacy show low reliabilities to the latent variable of stress perception. On the other side, items of helplessness and psychosocial working condition still show stable effects to their latent variables. Due to the little contribution of the dimension of low self-efficacy, M5 shows a similar situation with M3 that the standardized regression weights of working condition to stress perception, psychosocial working condition to needle-stick injury, and stress perception to needle-stick are 0.78, 0.39, and 0.32, respectively (*p* < 0.05). For the multi-group analysis, the effect of psychosocial working conditions on needle-stick injury (0.46, *p* < 0.05) is greater than that of stress perception (0.24, *p* < 0.05) among young employees aged 30 or younger, and such situation exerts oppositely among older employees. Nursing employees show higher regression weight from psychosocial working condition to stress perception 0.81 (*p* < 0.05), and less psychosocial working condition-stress perception effect 0.32 (*p* < 0.05) (See Table 4 and Fig. 1 for details).

**Table 4:** Standardized Path Coefficients of the structural model for the whole Sample and for the Multi-groups Separately

Variable		W to HPL	W to N	HPL to N	Model compare
Whole sample		0.78^**^	0.40^**^	0.31^**^	*p* < .001
Gender	Male	0.75^**^	0.41^**^	0.32^**^	*p* = .929
	Female	0.80^**^	0.40^**^	0.30^**^	
Age group	≤30 yrs.	0.80^**^	0.46^**^	0.24^**^	*p* < .001
	31–40 yrs.	0.82^**^	0.35^**^	0.38^**^	
	≥41 yrs.	0.69^**^	0.32^**^	0.34^**^	
Position	Physician	0.77^**^	0.47^**^	0.27^**^	*p* < .001
	Nurse	0.81^**^	0.32^**^	0.37^**^	
	Technician	0.66^**^	0.22^**^	0.33^**^	
Variable		W to LSE	W to N	LSE to N	Model compare
Whole sample		0.10^*^	0.63^**^	0.06^*^	*p* < .001
Gender	Male	0.06	0.64^**^	0.01	*p* = .095
	Female	0.12^**^	0.63^**^	0.08^*^	
Age group	≤30 yrs.	0.04	0.58^**^	0.07^*^	*p* = .012
	31–40 yrs.	0.16^*^	0.66^**^	0.01	
	≥41 yrs.	0.03	0.60^**^	0.09^*^	
Position	Physician	0.21^**^	0.59^**^	0.02	*p* < .001
	Nurse	0.03	0.62^**^	0.05	
	Technician	0.09^**^	0.56^**^	0.05	
Variable		W to SP	W to N	SP to N	Model compare
Whole sample		0.78^**^	0.39^**^	0.32^**^	*p* < .001
Gender	Male	0.75^**^	0.40^**^	0.33^**^	*p* = .933
	Female	0.80^**^	0.40^**^	0.31^**^	
Age group	≤30 yrs.	0.80^**^	0.46^**^	0.24^**^	*p* < .001
	31–40 yrs.	0.82^**^	0.35^**^	0.38^**^	
	≥41 yrs.	0.68^**^	0.32^**^	0.34^**^	
Position	Physician	0.77^**^	0.47^**^	0.28^**^	*p* < .001
	Nurse	0.81^**^	0.32^**^	0.37^**^	
	Technician	0.66^**^	0.22^**^	0.33^**^	

*W* = psychosocial working conditions, *HPL* helplessness, *LSE* low self-efficacy, *SP* stress perception, *N* needle-stick; *: *p* < 0.05; **: *p* < 0.01

### The standardized direct and indirect effects between analyzing variables

Direct and Indirect Effects were detected under the structural equation model between psychosocial working condition, stress perception, and needle-stick injury using bootstrap method. Results show that the direct effects of helplessness, low self-efficacy, and total stress perception on needle-stick injury are 0.31 (95%CI: 0.23 to 0.39), 0.06 (95%CI: 0.02 to 0.11), and 0.32 (95%CI: 0.22 to 0.39), respectively. And the direct effects of psychosocial working conditions on needle-stick injury in M3, M4, M5 models are 0.40 (95%CI: 0.32 to 0.47), 0.63 (95%CI: 0.59 to 0.66), and 0.39 (95%CI: 0.32 to 0.48), respectively. The indirect effects of psychosocial working conditions on needle-stick injury are helplessness, low self-efficacy, and total stress perception are 0.24 (95%CI: 0.18 to 0.30), 0.01 (95%CI: − 0.01 to 0.02), and 0.25 (95%CI: 0.19 to 0.31), respectively (See Table 5 for details).

**Table 5:** The Standardized Direct and Indirect Effects of the variables in the study

Variables		Effect	Bias-corrected 95%CI	*p*-value
Total effect				
Working condition(M3)	Needle-stick	0.64	0.60~0.67	<.001
Working condition(M4)	Needle-stick	0.63	0.59~0.66	<.001
Working condition(M5)	Needle-stick	0.64	0.60~0.67	<.001
Direct effect				
Working condition(M3)	Needle-stick	0.40	0.32~0.47	<.001
Working condition(M4)	Needle-stick	0.63	0.59~0.66	<.001
Working condition(M5)	Needle-stick	0.39	0.32~0.48	.040
Helplessness	Needle-stick	0.31	0.23~0.39	<.001
Low self-efficacy	Needle-stick	0.06	0.02~0.11	.010
Stress perception	Needle-stick	0.32	0.22~0.39	.020
Working condition(M3)	Helplessness	0.78	0.75~0.81	<.001
Working condition(M4)	Low self-efficacy	0.10	0.03~0.16	.004
Working condition(M5)	Stress perception	0.78	0.75~0.81	<.001
Indirect effect	Mediator		Needle-stick	
Working condition	Helplessness	0.24	0.18~0.30	<.001
	Low self-efficacy	0.01	−0.01~0.02	.070
	Stress perception	0.25	0.19~0.31	.015

## Discussion

Of the 1956 total participants, 900 (46.01%) had experienced at least one incident of needle-stick injury during the past year. For the valid samples as a whole, the prevalence of needle-stick injury during past year is 1.19 ± 1.75 times on average, which increases to 2.58 ± 1.76 times among those which have occurred at least once. According to the results of the correlation analyses, most correlation coefficients between psychosocial working conditions, stress perception, and needle-stick injury are of statistical significance. Needle-stick injury is positively correlated to job demand, job effort, over-commitment, helplessness, low self-efficacy, and total stress perception. And job control, social support, and job reward are negatively correlated to needle-stick injury. These results are primarily in line with the mainstream theoretical conclusions and previous studies [19, 33, 34]. Two recent studies show varied results on this topic. Mehrdad evaluated the prevalence of blood-borne exposure and psychosocial factors at work among nurses in Iran and found that 58.1% reported needle-stick injury, and those with middle- to high-level of stress had higher crude and adjusted odds than those with lower stress for all kinds of job injury [35]. However, Loerbroks concluded that only quantitative demands at work were associated with a slightly increased risk of subsequent sharp injury among nurses in a prospective study [36]. By contrast, the experience of sharp injury among nurses may predict less favorable perceptions of psychosocial work characteristics [36].

The rudimentary results above provide a blurred conclusion that there exist certain associations between psychosocial working conditions, stress perception, and incidents of needle-stick injury; however, how they are associated with each other will be addressed through the three hypotheses: 1) psychosocial working conditions directly relate to stress perception and employees’ experiences of needle-stick injury; 2) stress perception directly relates to the prevalence of needle-stick injury; 3) stress perception can mediate the relationship between psychosocial working conditions and needle-stick injury.

After the analyses of variable correlation and internal consistency, structural equation model tests were further conducted for the exploration of the three hypotheses. The results show that models of study hypotheses have satisfactory global goodness of fit. In addition, the direct effects of psychosocial working condition on helplessness, low self-efficacy, or total stress perception are 0.78, 0.10, or 0.78, respectively. Even though the coefficients have statistical significance, it is easy to conclude that a much stronger relationship exists between psychosocial working conditions and the feeling of helplessness, while psychosocial working conditions seems to have little effect on low self-efficacy. Since total stress perception is calculated by helplessness and low self-efficacy, the effect of psychosocial working conditions is largely assumed to be helplessness, which is in line with previous studies [37–39]. The direct effects of psychosocial working conditions on needle-stick injury in M3, M4, M5 models are 0.40 (95%CI: 0.32 to 0.47), 0.63 (95%CI: 0.59 to 0.66), and 0.39 (95%CI: 0.32 to 0.48), respectively, suggesting that stressful psychosocial working conditions such as high job demand, effort, over-commitment, and low job control, support, and reward are positively related to more incidents of needle-stick injury at work. Thus, Hypothesis 1 is supported.

The results show that the direct effects of helplessness, low self-efficacy, and total stress perception on needle-stick injury are 0.31 (95%CI: 0.23 to 0.39), 0.06 (95%CI: 0.02 to 0.11), and 0.32 (95%CI: 0.22 to 0.39), respectively. The effect of perceived stress on needle-stick injury is also largely performed by the dimension of helplessness. Similar phenomena appear in that the direct effect between low self-efficacy and needle-stick injury is only 0.06 (95%CI: 0.02~0.11). As positively measured in the PSS-10, the dimension of self-efficacy has been suggested not to be used independently by its author [40, 41]. Indeed, it is supposed that positive measurement accounts for a relatively small part of stress perception as a whole, e.g., Wang announced that dimension of self-efficacy only accounted for 14.80% of the variance in exploratory factor analysis of PSS-10, while perceived helplessness accounted for 48.9% [38]. However, some researchers still suggest introducing a two-dimension framework of analysis when using the scale of PSS-10 [27]. In the present study, we followed the latter suggestion in order to specify the effects between our study variables [37]. Nevertheless, the global stress perception is confirmed to be a significant predictor for events of sharp injury among medical workers [42], meaning that higher global stress perception certainly occurs alongside more incidents of needle-stick injury among healthcare workers. Therefore, Hypothesis 2 is also largely supported.

Hypothesis 3 proposed that stress perception may mediate the relationship between psychosocial working conditions and needle-stick injury, which is supported by the present results. Mediation analysis shows that in addition to the direct association with needle-stick injury, stress perception also has mediation effects. As is shown in Table 5, the total effect of psychosocial working conditions on needle-stick injury is stable, around 0.64, while mediation effects are 0.24, 0.01, or 0.25, indicating that stress perception, as the transformational embodiment of psychosocial working conditions mentally plays an important role in the occurrence of workplace injury, accounting for over one-third of the total effects of psychosocial working conditions on incidents of needle-stick injury. And the effective component within total stress perception is the negatively measured dimension: perceived helplessness. In other words, psychosocial working conditions still exerts a large proportion of total effects on needle-stick injury directly, while over one-third is affected through stress perception (the feeling of helplessness). Consequently, Hypothesis 3 is supported.

The results of the chi-squared test among the sub-groups of the three covariance show that young female nurses are at greater risk for needle-stick injury. Such situations have also been concluded by the multi-group analysis of the following structural equation model. For a deeper consideration of such phenomenon, female employees have always been supposed to possess higher levels of work-family conflict (less social support), more physical/mental demands (higher job demands) compared to male employees [43, 44]; younger workers are often supposed to have less experiences/skills (low job control), more monotonous ground work (higher demand), and less job reward [45]; and compared to technicians and physicians, nurses have more opportunity to access sharp instruments, and their heavy work load also relates to higher job demands and effort [46, 47]. In other words, these demographic characteristics, which often go alone with risking psychosocial working conditions are more susceptible to working injury under the circumstance of occupational stress [47].

The present study detected the relationship between stressful working conditions (stressor at work), personal stress perception (cognition difference), and prevalence of needle-stick injury (behavioral difference). Results show that more perceived stress always accompanies stressful working conditions, leading to more events of sharp injury at work. Consequently, psychosocial working conditions should be improved for the health of both healthcare workers and the organization. In addition, mediating analyses show that different stress levels would be perceived when different individuals face similar stressful conditions. Then, strategies of stress and behavior intervention should also focus on individual character and cognitive ability so as to yield satisfactory results.

The present study was based on self-report questionnaires, which might lead to the subjective bias. Nevertheless, the consistency of our findings under the theory, together with the acceptable sample size, suggests that common-method bias is not a major drawback of our study. Moreover, because of the limited availability of study samples, we only collected information from employees of first-class hospitals in Shanghai, which obviously had more prone to have better working conditions than lower class hospitals and hence the results could be underestimated. It might restrict the generalizability or external validation of our results. In future studies, a larger sample size and wider population from various regions should certainly be considered. And our data in this study was originated from a cross-sectional sample. Consequently, inferences on causality can only be made on the basis of prior conceptual assumptions.

## Conclusion

The present study focuses on exploring direct and indirect effects between psychosocial working condition and needle-stick injury. Through an analysis of the structural equation model, mediation effects analysis and other statistical procedures, the following hypotheses have been tested: 1) psychosocial working conditions directly relate to stress perception and employees’ experience of needle-stick injury; 2) stress perception directly relates to the prevalence of needle-stick; 3) stress perception can mediate the relationship between psychosocial working conditions and needle-stick injury. Psychosocial working conditions still comprise a large proportion of total effects on needle-stick injury, while over one–third of the correlative effect is affected through stress perception, and the effective component within total stress perception is the negatively measured dimension, perceived helplessness.

## Additional file

Additional file 1: Survey questionnaire. The questionnaire includes 3 sections that collect general information (Section 1: gender, age group, job position), Psychosocial working conditions (Section 2: JCQ, ERIQ), Stress perception (Section 3: PSS-10), and events of needle-stick injury (Section 3) from study participants. (DOCX 25 kb)

## Data Availability

Original data are available on request. These were stored on password-protected computers at Beijing Prevention and Treatment Hospital of Occupational Disease for Chemical Industry, China. Readers who wish to gain access to the data can write to the corresponding author.

## Abbreviations

AGFI: Adjustment fitting goodness indicator
CI: Confidence intervals
CMIN/DF: The minimum discrepancy
DCS: Job demand-control-social support model
ERI: Effort-reward imbalance model
ERIQ: Effort-Reward Imbalance Questionnaire
HBV: Hepatitis B virus
HCV: Hepatitis C virus
HIV: Human Immunodeficiency Virus
HPL: Helplessness
IFI: Incremental fit indicator
JCQ: Job Content Questionnaire
LSE: Low self-efficacy
N: Needle-stick
NNFI: Non-normalized Fit Index
PSS-10: Perceived Stress Scale
RMSEA: Root mean square error of approximation
SP: Stress perception
W: Psychosocial working conditions
