# Supplementary material for: Relationship between psychosocial working conditions, stress perception, and needle-stick injury among healthcare workers in Shanghai
Source: BMC Public Health. 2019 Jul 4;19:874. doi: 10.1186/s12889-019-7181-7 (PMC6610837; doi:10.1186/s12889-019-7181-7)
Supplement: Supplementary file 1 — Survey questionnaire. The questionnaire includes 3 sections that collect general information (Section 1: gender, age group, job position), Psychosocial working conditions (Section 2: JCQ, ERIQ), Stress perception (Section 3: PSS-10), and events of needle-stick injury (Section 3) from study participants. (DOCX 25 kb) [file 12889_2019_7181_MOESM1_ESM.docx]

**We kindly ask you to answer the following questions and statements. By doing so**

**you contribute to a better scientific understanding of associations between modern**

**working life and health. Thank you in advance!**

**Section 1 General Information**

1. Name:_________
2. What is your Gender

(1) male (2) female

3. Which Age Group do you belong to?

(1) 30 years old or below (2) 31 to 40 years old (3) 41 years old or above

4. What is your Job Position during the past one year?

(1) physician (2) nurse (3) technician

**Section 2 Psychosocial Working Conditions**

1. For each of the following statements, please indicate whether you agree or disagree

with it. please also indicate how much you are generally distressed by this situation (1 = I disagree not at all; 2 = I somewhat disagree; 3 = I basically agree; 4 = I agree; 5= I strongly agree). Thank you for answering all statements.

| JCQ | **1** strongly  disagree | **2** disagree | **3** basically agree | **4**  agree | **5** strongly  agree |
| --- | --- | --- | --- | --- | --- |
| 1.My job requires working very fast | 1 | 2 | 3 | 4 | 5 |
| 2.My job requires working very hard | 1 | 2 | 3 | 4 | 5 |
| 3.I am asked to do an excessive amount of work | 1 | 2 | 3 | 4 | 5 |
| 4.I do not have enough time to get the job done | 1 | 2 | 3 | 4 | 5 |
| 5.I suffer from conflicting demands others make | 1 | 2 | 3 | 4 | 5 |
| 6.On my job, I am given a lot of freedom  to decide how I do my work | 1 | 2 | 3 | 4 | 5 |
| 7.My job allows me to make a lot of decisions on my own | 1 | 2 | 3 | 4 | 5 |
| 8.My job requires that I learn new things | 1 | 2 | 3 | 4 | 5 |
| 9.My job requires a high level of skill | 1 | 2 | 3 | 4 | 5 |
| 10.My job requires me to be creative | 1 | 2 | 3 | 4 | 5 |
| 11.My job involves a lot of repetitive work | 1 | 2 | 3 | 4 | 5 |
| 12.My family support my job | 1 | 2 | 3 | 4 | 5 |
| 13.People I work with are helpful in getting the job done | 1 | 2 | 3 | 4 | 5 |
| 14.People I work with are friendly | 1 | 2 | 3 | 4 | 5 |
| 15.My supervisor is helpful in getting the job done | 1 | 2 | 3 | 4 | 5 |
| 16.My supervisor is successful in  getting people to work together | 1 | 2 | 3 | 4 | 5 |

1. For each of the following statements, please indicate whether you agree or disagree with it. please also indicate how much you are generally distressed by this situation (1 = I disagree not at all; 2 = I somewhat disagree; 3 = I basically agree; 4 = I agree; 5= I strongly agree). Thank you for answering all statements.

| ERI | **1** strongly  disagree | **2** disagree | **3** basically agree | **4**  agree | **5**  strongly  agree |
| --- | --- | --- | --- | --- | --- |
| 1.I have constant time pressure due to a heavy work load | 1 | 2 | 3 | 4 | 5 |
| 2.I have many interruptions and disturbances in my job | 1 | 2 | 3 | 4 | 5 |
| 3.I have a lot of responsibility in my job | 1 | 2 | 3 | 4 | 5 |
| 4.I am often pressured to work overtime | 1 | 2 | 3 | 4 | 5 |
| 5.My job is physically demanding | 1 | 2 | 3 | 4 | 5 |
| 6.Over the past years, my job has become more and more demanding | 1 | 2 | 3 | 4 | 5 |
| 7.I do not receive the respect I deserve from my superiors | 1 | 2 | 3 | 4 | 5 |
| 8.I do not receive the respect I deserve from my colleagues | 1 | 2 | 3 | 4 | 5 |
| 9.I experience inadequate support in difficult situations | 1 | 2 | 3 | 4 | 5 |
| 10.I am treated unfairly at work | 1 | 2 | 3 | 4 | 5 |
| 11.My job promotion prospects are poor | 1 | 2 | 3 | 4 | 5 |
| 12.I have experienced or I expect to experience an undesirable change in my work situation | 1 | 2 | 3 | 4 | 5 |
| 13.My job security is poor | 1 | 2 | 3 | 4 | 5 |
| 14.My current occupational position inadequately reflects my education and training | 1 | 2 | 3 | 4 | 5 |
| 15.Considering all my efforts and achievements, I receive the respect and prestige I deserve at work | 1 | 2 | 3 | 4 | 5 |
| 16.Considering all my efforts and achievements, my work prospects are inadequate. | 1 | 2 | 3 | 4 | 5 |
| 17.Considering all my efforts and achievements, my salary / income is inadequate | 1 | 2 | 3 | 4 | 5 |
| 18.I get easily overwhelmed by time pressures at work | 1 | 2 | 3 | 4 | 5 |
| 19.I start thinking about work problems as soon as I get up in the morning | 1 | 2 | 3 | 4 | 5 |
| 20.People close to me say I sacrifice too much for my job | 1 | 2 | 3 | 4 | 5 |
| 21.Work is usually still on my mind when I go to bed | 1 | 2 | 3 | 4 | 5 |
| 22. If I put off something that needs to be done today, I'll have trouble sleeping at night | 1 | 2 | 3 | 4 | 5 |

**Section 3 Stress perception & Needle-stick injury**

1. For each of the following statements, please indicate how often you experience with it. (1 = Never; 2 = Rarely; 3 = Sometimes; 4 = often; 5= Always). Thank you for answering all statements.

| PSS-10 | **1**  **Never** | **2 Rarely** | **3 Sometimes** | **4**  **Often** | **5**  **Always** |
| --- | --- | --- | --- | --- | --- |
| 1.In the last month, how often have you been upset because of something that happened unexpectedly? | 1 | 2 | 3 | 4 | 5 |
| 2.In the last month, how often have you felt that you were unable to control the important things in your life? | 1 | 2 | 3 | 4 | 5 |
| 3.In the last month, how often have you felt nervous and stressed? | 1 | 2 | 3 | 4 | 5 |
| 4.In the last month, how often have you been confident about your ability to handle  your personal problems? | 1 | 2 | 3 | 4 | 5 |
| 5.In the last month, how often have you felt that things were going your way? | 1 | 2 | 3 | 4 | 5 |
| 6.In the last month, how often have you found that you could not cope with all  the things that you had to do? | 1 | 2 | 3 | 4 | 5 |
| 7.In the last month, how often have you been able to control irritations in your  life? | 1 | 2 | 3 | 4 | 5 |
| 8.In the last month, how often have you dealt successfully with irritating life  hassles? | 1 | 2 | 3 | 4 | 5 |
| 9.In the last month, how often have you  been angered because of things that  happened that were outside of your  control? | 1 | 2 | 3 | 4 | 5 |
| 10.In the last month, how often have you felt difficulties were piling up so high  that you could not overcome them? | 1 | 2 | 3 | 4 | 5 |

2. During the past one year, how many times did you experience a needle-stick injury?

Answer: ___________ times.
